# Toscana Virus and Acute Meningitis, France

**DOI:** 10.3201/eid1105.041122

**Published:** 2005-05

**Authors:** Christophe N. Peyrefitte, Ivan Devetakov, Boris Pastorino, Laurent Villeneuve, Mael Bessaud, Philippe Stolidi, Jerome Depaquit, Laurence Segura, Patrick Gravier, Fabienne Tock, Francoise Durand, Jean-Paul Vagneur, Hugues J. Tolou, Marc Grandadam

**Affiliations:** * Laboratoire Associé au Centre National de Référence des Arbovirus, Marseille, France;; † Centre Hospitalier Edmond Garcin, Aubagne, France;; ‡ Faculté de Pharmacie, Reims, France

**Keywords:** Toscana virus, acute meningitis, French case

**To the Editor:** Sandfly fever Naples virus, Sandfly fever Sicilian virus, and Toscana virus (family *Bunyaviridae*, genus *Phlebovirus*) have been recognized as etiologic agents of human illnesses in European countries bordering the Mediterranean Sea. These viruses are responsible for rapidly resolving diseases with nonspecific symptoms such as fever and myalgia. However, infection with Toscana virus may involve the central nervous system; severity may range from aseptic meningitis to meningoencephalitis (1). In most cases, illnesses caused by Toscana virus mimics a flulike syndrome with fever, photophobia, headache, red eyes, and stiff neck. Recently, 2 cases of Toscana virus meningoencephalitis in patients with unusual symptoms and life-threatening complications were described in Italy (2). However, sequelae have never been reported.

Toscana virus infection is now epidemic in Italy and Spain (1,3). Furthermore, sporadic cases have been reported in travelers returning from Italy, Spain, Greece, Portugal, and the South of France (4–6). The epidemiology of Toscana virus in France is still unknown. Although infections with this virus have been diagnosed by serologic tests in French patients and in tourists residing in southeastern France, this pathogen has reportedly never been isolated in France (7,8). Here we describe the clinical and biologic features of autochthonous meningitis due to Toscana virus.

On July 9, 2004, a 57-year-old woman who had never left the southeastern coast of France reported malaise and vomiting. On hospital admission, her body temperature was 38.5°C, and clinical examination showed photophobia and stiff neck. Skin and abdomen were normal. Cardiopulmonary and neurologic functions were also normal. Analysis of hematologic and biochemical blood tests revealed mild hyperglycemia (6.88 mmol/L) and elevated γ-glutamyltransferase (104 IU/L) and C-reactive protein levels (57 mg/L). Cerebrospinal fluid (CSF) analysis showed 3,500 leukocytes/μL (70% lymphocytes, 30% neutrophils), and glucose and protein levels of 2.5 mmol/L and 2.749 mg/L, respectively. Blood and CSF cultures were bacteriologically sterile. Polymerase chain reaction (PCR) assays of CSF for herpes simplex virus were also negative. The patient received intravenous amoxicillin and acyclovir for 3 days. The patient recovered after 6 days without sequelae.

Serum and CSF samples collected during the acute phase were tested for immunoglobulin (Ig)M and IgG antibodies to a battery of arboviruses. These samples contained no antibodies (optical density [OD] ratio <1.5) to flaviviruses, dengue virus, West Nile virus, and tickborne encephalitis, Tahyna virus, or Sandfly fever Sicilian virus (Table). However, the IgM OD ratios (≥2.5) obtained against Toscana virus antigen were high. A second serum sample tested 1 month later showed seroconversion to Toscana virus with OD ratios >3 for both IgM and IgG (Table).

**Table:** Arbovirus antibody investigation of samples*

Viral antigens	CSF†		Serum 1†		Serum 2‡	
	IgM§	IgG¶	IgM	IgG	IgM	IgG
Dengue	1.16	1.08	1.06	1.06	1.32	0.94
West Nile	1.06	0.92	0.96	1.03	1.27	1.29
Toscana	2.84	0.97	2.50	0.94	**48.72**	**3.48**
Sandfly fever Sicilian	0.98	0.96	0.96	0.96	1.20	0.86
Tickborne encephalitis (Langat)	0.88	1.15	1.09	0.96	1.22	0.74
Tahyna	0.98	0.94	0.96	1.00	1.20	1.17

*CSF, cerebrospinal fluid; Ig, immunoglobulin; MAC-ELISA, immunoglobulin M antigen capture enzyme-linked immunosorbent assay. Values are the ratio OD(viral antigen)/OD(control antigen). Samples were considered positive if the ratio is over 3. **Bold** values indicate positive results. †CSF and serum obtained at the onset of the disease. ‡Serum obtained 30 days after the onset of the disease. §MAC-ELISA. ¶Sandwich-ELISA.

Virus isolation was attempted by incubation of peripheral blood mononuclear cells and CSF collected on the day of onset with C6/36 (*Aedes albopictus*) and Vero (E6 clone) monolayers. Toscana virus was found only on Vero cells by indirect immunofluorescence by using mouse hyperimmune ascitic fluid against Toscana virus. In contrast, no fluorescence was found by using mouse hyperimmune ascitic fluids against Rift Valley fever and Sandfly fever Sicilian virus.

S segment of Toscana virus genome was partly amplified from culture supernatants by reverse-transcription PCR and sequenced (9). Nucleotide and peptide sequences obtained (GenBank accession no. AY766034) displayed 87% and 100% identity, respectively, with Toscana virus sequences available on GenBank database, thus confirming the infection by Toscana virus.

Toscana virus, transmitted to humans by *Phlebotomus* vectors, has been recognized as a major cause of aseptic meningitis in Italy and Spain. *P. perniciosus*, proven to be a vector of Toscana virus (10), is abundant along the French Mediterranean coast. The isolation of an autochthonous Toscana virus strain shows that the conditions of an efficient transmission cycle were combined in southern France. Until now, human infection by Toscana virus was fortuitously detected by serologic means, suggesting that subclinical infection may also occur (8). Thus, Toscana virus infection in France likely has been underestimated. Moreover, meningitis caused by Toscana virus has been underestimated and other diseases caused by Toscana virus may have also been underestimated. The requirement for virus growth in cell culture delays a diagnosis based on viral isolation, which is limited by the transitory presence of the virus in blood or CSF. As reported here, Toscana virus infection was only confirmed after the patient relapsed. Considering that signs and symptoms of Toscana virus meningitis are not pathognomic, this case highlights the need for rapid and specific diagnostic tools, such as PCR assays, to identify infections caused by Toscana virus and other neurotropic viral agents. Moreover, a systematic serologic study of recovered meningitis patients may help to better characterize viral meningitis of unknown etiology.

Finally, this work suggests that, in addition to West Nile virus, Toscana virus should now be considered as a potential etiologic agent of acute meningitis in the southeastern part of France. Entomologic and epidemiologic surveys, however, will have to be conducted in the near future to determine the risk for the people living in that area.
