# Correction: Vol. 11, No. 5

**DOI:** 10.3201/eid1106.C11106

**Published:** 2005-06

**Keywords:** Correction, errata, erratum

In “Adenovirus Type 7 Peptide Diversity during Outbreak, Korea, 1995–2000,” by Eun Hwa Choi et al., errors occurred. In

Table 2, column 3, amino acid sequences should be identified at position 197.

Also, Figures 1 and 2 in this article do not show all information. The online figures correctly represent the findings.

The corrected article appears online at http://wwwnc.cdc.gov/eid/article/11/5/04-1211_article.htm.

We regret any confusion these errors may have caused.

